# Temperature Effects on the Virulence and Horizontal Transmission of *Beauveria bassiana* Against *Zeugodacus cucurbitae* with SIR Model Analysis

**DOI:** 10.3390/insects17050475

**Published:** 2026-05-05

**Authors:** Ying Fu, Jingpeng Xie, Zhongshi Zhou, Lang Fu, Jian Wen, Fengqin Cao

**Affiliations:** 1 School of Tropical Agriculture and Forestry, Hainan University, Haikou 570228, China; 18703987713@163.com (Y.F.); xiejp20240612@163.com (J.X.); 2 State Key Laboratory for Biology of Plant Diseases and Insect Pests, Institute of Plant Protection, Chinese Academy of Agricultural Sciences, Beijing 100193, China; zhouzhongshi@caas.cn; 3 National Nanfan Research Institute, Chinese Academy of Agricultural Sciences, Sanya 572019, China; 4 Hunan Horticulture Research Institute, Hunan Academy of Agricultural Sciences, Changsha 410125, China; m18305918768@163.com

**Keywords:** tephritid fruit fly, entomopathogenic fungi, temperature effect, horizontal transmission, model prediction

## Abstract

Entomopathogenic fungi are vital components of integrated pest management; however, environmental temperature is one of the key factors that limits their individual-level virulence, population-level transmission dynamics, and field efficacy. In this study, we isolated an indigenous fungal strain from a naturally infected *Zeugodacus cucurbitae* cadaver, and evaluated its biological characteristics, virulence, and horizontal transmission efficiency against *Z. cucurbitae* across 20–35 °C. A temperature-driven Susceptible–Infected–Removed (SIR) epidemiological model was developed to simulate infection dynamics and predict its epizootic potential. Morphological and molecular (ITS) analyses identified the isolate as *Beauveria bassiana*, designated as strain WZS5. WZS5 exhibited notable thermotolerance. At 30 °C, the strain displayed a short median germination time (7.7 h), high sporulation yield (1.1 × 10^8^ conidia mL^−1^), and fast radial growth (4.8 mm d^−1^). Additionally, it showed substantial virulence with a median lethal concentration (LC_50_) of 1.32 × 10^7^ conidia mL^−1^ and a median lethal time (LT_50_) of 5.28 days at a concentration of 1.0 × 10^8^ conidia mL^−1^. Baseline biological activity was maintained even at 35 °C. At 30 °C, horizontal transmission was effective, yielding a cadaver sporulation rate of approximately 70.0%, a 4.0-day sporulation lag, and viable F_1_ conidia (88.6% germination). The SIR model adequately captured these dynamics (r = 0.919), predicting potential epizootic spread at 30 °C with a basic reproduction number (R_0_) of 1.90. This predictive framework quantifies temperature thresholds for fungal epizootics, providing valuable ecological insights for managing *Z. cucurbitae* in tropical and subtropical regions.

## Simple Summary

The melon fly, *Zeugodacus cucurbitae*, is a quarantine pest of global importance that infests a wide range of economically important crops, including bitter gourd and cucumber. Control of this pest has long relied heavily on broad-spectrum chemical insecticides. However, this approach has raised major ecological concerns. Therefore, introducing biocontrol agents with relatively low ecological risk and good compatibility with natural ecological processes may help maintain ecological balance in the field. In this study, we isolated an indigenous fungal strain from a naturally infected *Z. cucurbitae* cadaver. We evaluated its biological characteristics, virulence, and horizontal transmission efficiency against *Z. cucurbitae* across 20–35 °C. A temperature-driven Susceptible–Infected–Removed (SIR) epidemiological model was developed to simulate infection dynamics and predict its epizootic potential. These findings suggest that *B. bassiana* WZS5 has potential not only as a direct mycoinsecticide but also as a longer-term ecological regulator of field populations, and a cost-effective integrated management strategy for *Z. cucurbitae* in tropical and subtropical regions.

## 1. Introduction

In agroecosystems, integrated pest management is increasingly adopting an ecosystem-based approach. Understanding the interactions among pests, natural enemies, and abiotic environmental factors is essential for developing sustainable pest control strategies [1,2,3]. The melon fly, *Zeugodacus cucurbitae*, is a quarantine pest of global importance that infests a wide range of economically important crops, including bitter gourd and cucumber [4,5,6]. Control of this pest has long relied heavily on broad-spectrum chemical insecticides. However, this approach has raised major ecological concerns. On the one hand, field populations of *Z. cucurbitae* have developed notable resistance to spinosad and several pyrethroid insecticides [7,8]. On the other hand, pesticide application has negatively affected non-target organisms enemies in agroecosystems, such as *Fopius arisanus*, a parasitoid wasp that contributes to the natural control of *Z. cucurbitae*, thereby disrupting ecological interactions in the field [9]. Therefore, introducing biocontrol agents with relatively low ecological risk and good compatibility with natural ecological processes may help maintain ecological balance in the field.

Entomopathogenic fungi are recognized as natural regulators of arthropod populations and can contribute to sustainable pest management [10,11]. Among them, *Beauveria bassiana* infects insects by adhering to the host cuticle, germinating, penetrating the integument, and secreting enzymes and toxins, eventually colonizing the hemocoel and causing host death [12]. Unlike entomopathogens that must be ingested to act, *B. bassiana* relies on a contact-mediated cuticular infection mechanism, making it potentially suitable for controlling adult tephritid flies [13,14]. Nevertheless, when laboratory-screened biocontrol fungi are applied under field conditions, their efficacy is often constrained by abiotic ecological factors [15], among which ambient temperature is considered one of the most critical [16].

Temperature affects fungal physiological processes such as conidial germination, radial growth, and sporulation capacity. It also influences the thermoregulatory behavior and immune responses of the host insect. Together, these factors shape the outcome of host–pathogen interactions. [17,18]. Several commercial strains of *B. bassiana* show relatively high activity at moderate temperatures of 20–25 °C, but their performance tends to decline at higher temperatures. For example, Bugeme et al. [19]. (2008) found in studies on the two-spotted spider mite (*Tetranychus urticae*) that conidial germination was inhibited and pathogenicity decreased when ambient temperature exceeded 30 °C. Velavan et al. [20] (2022) further suggested that fungal genotype and thermal tolerance are often associated with the climatic microhabitat of the isolation site. *Zeugodacus cucurbitae* occurs predominantly in tropical and subtropical regions [6], where high temperatures persist throughout summer and autumn. Therefore, screening indigenous strains from local high-temperature microhabitats and characterizing their thermal response profiles is important for improving ecological pest regulation in these regions.

In addition, evaluating the biocontrol potential of entomopathogenic fungi requires attention not only to individual-level virulence but also to horizontal transmission at the host population level. In natural ecosystems, cadavers of infected individuals can serve as inoculum sources by releasing secondary conidia that infect surrounding healthy conspecifics. Previous studies have documented horizontal transmission in tephritid pests: Meadow et al. [21] (2000) reported that *B. bassiana* could be transferred through contact among adult flies; Toledo et al. [22] (2007) observed secondary infections caused by infected cadavers in populations of the Mexican fruit fly (*Anastrepha ludens*); and Gálvez et al. [23] (2023) showed that *B. bassiana* conidia could be passed between individuals during courtship and mating in the Mediterranean fruit fly (*Ceratitis capitata*). To quantitatively analyze these transmission processes, epidemiological models such as the susceptible–infected–removed (SIR) model have been increasingly used [24,25,26]. By incorporating environmental temperature and contact transmission parameters, such models can help predict how environmental variation influences epizootic development. However, quantitative studies on how temperature regulates horizontal transmission of *B. bassiana* within *Z. cucurbitae* populations are still limited.

In November 2023, an indigenous *B. bassiana* strain, designated WZS5, was isolated and identified from a naturally infected *Z. cucurbitae* specimen collected in a cucurbit field with a mean daily temperature of approximately 30 °C in Wuzhishan City, Hainan Province, China. This study aimed to characterize the biological properties of strain WZS5, evaluate its biocontrol potential against *Z. cucurbitae*, assess its horizontal transmission efficiency within *Z. cucurbitae* cohorts and the sporulation capacity of infected cadavers, and examine the effects of temperature on these traits. A temperature-dependent SIR epidemiological model was also constructed for this host–pathogen system. This study provides preliminary evidence on the ecological mechanisms of fungal pest suppression in tropical environments and may inform the development of integrated field management strategies.

## 2. Materials and Methods

### 2.1. Insect Rearing and Establishment of a Standardized Colony

The initial *Zeugodacus cucurbitae* colony used in this study was obtained from the Chinese Academy of Tropical Agricultural Sciences, Haikou, China. The colony was maintained in the laboratory for more than 30 generations to establish a stable laboratory population. All rearing was conducted in a controlled-environment chamber (Mingtu Machinery, Changge, China) at 26 ± 1 °C, 65 ± 5% relative humidity (RH), and a 12L:12D photoperiod. Larvae were fed fresh zucchini. Zucchini pieces containing larvae were placed in plastic trays with a base layer of sand (approximately 30% moisture content) as a pupation substrate. Every three days, pupae were sifted out using a mesh sieve and transferred to plastic containers lined with moist filter paper, where they were allowed to emerge under the controlled conditions described above. Newly emerged adults were transferred to rearing cages and provided with a dry powder diet of yeast extract and glucose (1:3, *w*/*w*) along with sterile water. All bioassays used healthy adults (they exhibited normal locomotion, no visible wing deformities, and no signs of fungal infection at the time of collection) of uniform size from the same emergence cohort at 3 days post eclosion.

### 2.2. Field Collection and Identification of the Entomopathogenic Fungus

In November 2023, samples were collected from a cucurbit field in Wuzhishan City, Hainan Province (mean daily temperature approximately 30 °C, RH approximately 70%) using yellow sticky traps. Mycosed *Z. cucurbitae* cadavers were removed from the traps in a laminar flow cabinet, surface-sterilized with 0.5% sodium hypochlorite solution for 1 min, and rinsed three times with sterile water. Hyphal fragments from the cadaver surface were transferred onto potato dextrose agar (PDA) plates supplemented with 100 ug mL^−1^ ampicillin. Plates were incubated in darkness at 28 ± 1 °C for 3–5 days, and single colonies were subcultured onto fresh PDA plates two to three times to obtain a pure culture, designated WZS5. For molecular identification, genomic DNA was extracted using a commercial kit (Servicebio, Wuhan, China). The internal transcribed spacer (ITS) region was amplified using universal primers (F: 5′-CCGTGTTTCAAGACGGG-3′; R: 5′-CTTGGTCATTTAGAGGAAGTAA-3′). Each PCR reaction contained 12.5 μL of 2× Taq Master Mix, 1.0 μL of each primer, 2.0 μL of template DNA, and nuclease-free water to a final volume of 25 μL. The thermal cycling program was as follows: initial denaturation at 95 °C for 3 min; 32 cycles of 95 °C for 30 s, 55 °C for 30 s, and 72 °C for 1 min; and a final extension at 72 °C for 5 min. PCR products were sent to Wuhan Servicebio Technology Co., Ltd., Wuhan, China for sequencing. The resulting sequences were compared with those in the GenBank database using BLAST, and a phylogenetic tree was constructed.

### 2.3. Effects of Temperature on the Biological Characteristics of WZS5

Five temperature treatments (20, 25, 28, 30, and 35 °C) were established, and all tests were conducted on PDA medium. For conidial germination, a suspension of 1.0 × 10^6^ conidia mL^−1^ was prepared and inoculated onto a thin layer of PDA on glass slides, which were then incubated under moist conditions. Germination was assessed at 6, 12, 18, and 24 h after inoculation, with a conidium considered germinated when the germ tube length exceeded the spore radius. Four replicates were included per treatment, with 200 conidia examined per replicate. The time required for 50% germination (GT_50_) was calculated. For radial growth, mycelial plugs (5 mm in diameter) were placed at the center of PDA plates and incubated in darkness at each temperature. Colony diameters were measured daily for 14 consecutive days by measuring two perpendicular diameters, with ten replicates per treatment. For sporulation, colonies previously used for diameter measurements were cultured for a total of 15 days, after which three mycelial plugs (1 cm in diameter) were excised at equal distances 1 cm from the colony center, pooled, and suspended in 0.05% Tween-80. Conidia were then counted using a hemocytometer. Ten replicates were included per treatment.

### 2.4. Bioassay of Virulence as Affected by Temperature and Conidial Concentration

To determine the interactive effects of temperature and conidial concentration, a two-factor completely randomized design was used. Fresh conidia were harvested from 15-day-old PDA cultures and suspended in sterile water containing 0.05% Tween-80 to prepare five concentration levels: 1.0 × 10^5^, 1.0 × 10^6^, 1.0 × 10^7^, 1.0 × 10^8^, and 1.0 × 10^9^ conidia mL^−1^. Healthy *Z. cucurbitae* adults were selected and placed in stainless-steel mesh cages (20 × 20 × 20 cm), and 20 mL of the corresponding conidial suspension was evenly sprayed directly onto the adult flies inside the cage using a 50 mL spray bottle (Servicebio, Wuhan, China). Control groups were sprayed with an equal volume of sterile water containing 0.05% Tween-80. Each treatment combination consisted of 30 adults with four replicates. Ten minutes after treatment, insects were transferred to rearing containers with artificial diet and placed in controlled-environment chambers at the five temperature levels described above (20–35 °C), with RH maintained at 75 ± 5% under a 12L:12D photoperiod. Mortality was recorded daily for 30 consecutive days. Dead individuals were removed and placed in Petri dishes containing sterile moist filter paper (25 °C, RH > 75%) for 5–7 days; those exhibiting characteristic white hyphal growth were confirmed as deaths caused by fungal infection. Corrected mortality and cumulative mortality were calculated, and the median lethal concentration (LC_50_) and median lethal time (LT_50_) were estimated using probit analysis.

$$Corrected\;mortality\;(\%)=\frac{Mortality\;in\;treatment-Mortality\;in\;control}{\left(100-Mortality\;in\;control\right)}\times 100$$

### 2.5. Horizontal Transmission Within Population

To examine the epizootic potential of the entomopathogenic fungus within the host population, simulated contact transmission experiments were conducted at three representative temperatures: 20, 30, and 35 °C (these three temperatures were selected based on the bioassay results in Section 2.4). *Zeugodacus cucurbitae* adults were immersed in a 1.0 × 10^8^ conidia mL^−1^ suspension for 30 s to serve as infection donors and then kept under moist conditions at the corresponding temperature and 75 ± 5% RH for 24 h to allow conidial adhesion and initial germination. Donors were then mixed with healthy recipient adults at ratios of 1:1, 1:3, and 1:5 (infected: healthy) in mesh cages, with a total of 36 individuals per cage. Each mixed group was maintained at its assigned temperature, and total mortality of all adults within each cage was recorded daily for 30 days. Cadavers confirmed to have died from fungal infection in each temperature treatment were individually incubated under moist conditions, and the proportion producing new conidia (i.e., sporulation rate) was recorded. Among cadavers that successfully sporulated, conidia were washed off each cadaver three times, each with 5 mL of sterile water; the washings were pooled, and conidia were counted using a hemocytometer to determine per-cadaver conidial yield. The time from host death to the appearance of visible white mycelia on the cadaver surface (sporulation lag time) was also recorded. Additionally, the germination rates of primary (F_0_) and secondary (F_1_) conidia were calculated and compared. Five replicates were set up per treatment.

### 2.6. Construction of a Temperature-Dependent SIR Epidemiological Model

Based on classical infectious disease dynamics theory [26,27], the experimental *Z. cucurbitae* population was divided into three compartments, susceptible (*S*), infected and infectious (*I*), and removed (*R*) individuals, with total population size *N*. Assuming a closed population (*S* + *I* + *R* = *N*) and homogeneous random mixing, the horizontal transmission dynamics of the fungus were described by the following system of ordinary differential equations:

$$\frac{dS}{dt}=-\frac{\beta SI}{N}$$

$$\frac{dI}{dt}=\frac{\beta SI}{N}-\gamma I$$

$$\frac{dR}{dt}=\gamma I$$

where *β* represents the effective transmission coefficient (day^−1^), quantifying the rate at which an infected individual contacts and successfully infects susceptible individuals per unit of time; *γ* denotes the disease-induced removal rate (day^−1^), calculated based on the median lethal time (*LT*_50_) as:

$$\gamma =\frac{ln(2)}{LT_{50}}$$

The basic reproduction number (R_0_), indicating the epizootic potential, is defined as:

(1) $$R_{0}=\frac{\beta }{\gamma }$$

Model parameters were estimated from the horizontal transmission data obtained at each temperature using the non-linear least-squares method. The optimization objective was to minimize the residual sum of squares (RSS) between the observed cumulative mortality and the model predictions. Subsequently, regression analysis was employed to fit quadratic polynomial functions characterizing the relationship of both the transmission coefficient (*β*) and the removal rate (*γ*) with temperature, thereby facilitating the quantitative prediction of epizootic potential across the tested thermal range.

### 2.7. Data Analysis

All statistical analyses were performed using R software (4.5.0). Because some datasets did not meet the assumptions of normality or homogeneity of variance, non-parametric tests were used for group comparisons without prior data transformation.

The Kruskal–Wallis rank sum test was used to assess the effects of temperature on the following biological parameters: median germination time (GT_50_), daily radial growth rate, sporulation yield on plates, corrected mortality under different temperatures and conidial concentrations, corrected mortality under different temperatures and initial infection (I: H) ratios, cadaver sporulation rate, per-cadaver conidial yield, and sporulation lag time. When the Kruskal–Wallis test indicated significant differences (*p* < 0.05), pairwise comparisons were performed using the Wilcoxon rank sum test. To control the family-wise error rate, *p*-values from multiple comparisons were adjusted using the Bonferroni correction. The germination rates of parental (F_0_) and progeny (F_1_) conidia were compared using the Wilcoxon rank sum test (Mann–Whitney U test).

The median lethal concentration (LC_50_) and median lethal time (LT_50_), along with their 95% confidence intervals, were estimated using generalized linear models (GLMs) with a binomial error distribution and a probit link function. The probit regression model was defined as:

$$\mathrm{Probit}(p)=a+b\times {log}_{10}(x)$$

where *p* represents the probability of mortality, *a* is the intercept, *b* is the slope, and *x* denotes either conidial concentration (for LC_50_) or time in days (for LT_50_). The LC_50_ and LT_50_ values were calculated as:

$$LC_{50}\;\mathrm{or}\;LT_{50}=10^{-a/b}$$

Parameters of the SIR model, specifically the transmission coefficient (*β*) and removal rate (*γ*), were estimated by minimizing the residual sum of squares (RSS) between observed and predicted cumulative mortality using nonlinear least squares (Section 2.6). Model fit was evaluated by linear regression of observed versus predicted values and by the Pearson correlation coefficient (*r*). Quadratic regression was further used to quantify the relationships between model parameters (*β*, *γ*) and temperature. The significance level for all statistical tests was set at α = 0.05.

## 3. Results

### 3.1. Field Collection and Identification of the Entomopathogenic Fungus

**Figure 1:**
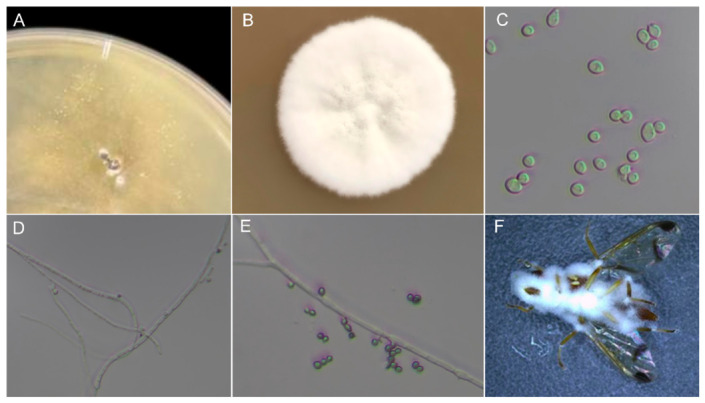
Morphological characteristics of *B. bassiana* isolated from naturally infected *Z. cucurbitae* cadavers. (**A**) Initial fungal outgrowth from field-collected cadaver on PDA; (**B**) Colony morphology on PDA after 14 days at 28 °C; (**C**) Conidia under light microscopy; (**D**) Vegetative hyphae; (**E**) Zigzag-shaped conidiogenous cells with conidia; (**F**) Artificially inoculated adult cadaver covered with white mycelia.

**Figure 2:**
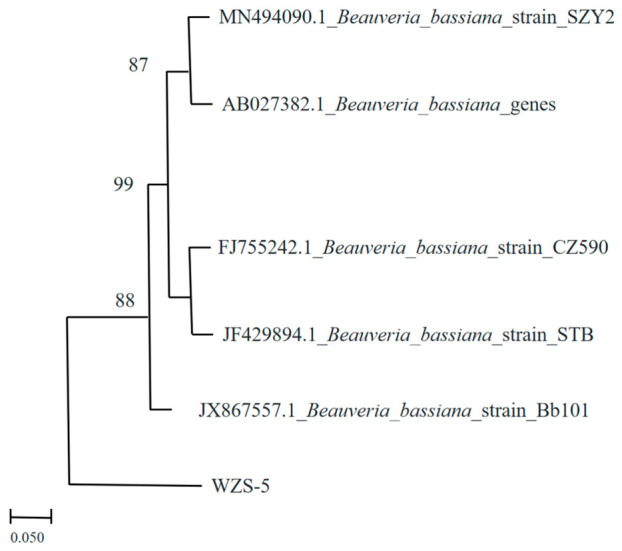
Phylogenetic tree showing the relationship between isolate WZS5 and related *B. bassiana* strains based on ITS sequence data. Bootstrap support values (%) are indicated at the nodes, and the scale bar represents 0.05 nucleotide substitutions per site.

A fungal pathogen was isolated from field-collected *Z. cucurbitae* cadavers displaying white muscardine symptoms (Figure 1A). The purified isolate produced circular, white to cream-colored, cottony-to-powdery colonies with abundant aerial mycelia and characteristic radial furrows on PDA after 14 days at 28 °C, with no diffusible pigment on the reverse (Figure 1B). Conidia were hyaline, single-celled, globose to subglobose, with smooth walls (Figure 1C). Vegetative hyphae were hyaline, septate, and irregularly branched (Figure 1D). Conidiogenous cells displayed the diagnostic zigzag-shaped (geniculate) rachis typical of *Beauveria*, producing conidia holoblastically in sympodial succession with denticulate scars on the elongating neck (Figure 1E) [28]. Artificial inoculation confirmed pathogenicity, as treated *Z. cucurbitae* adults became completely enveloped by dense white sporulating mycelia (Figure 1F). Based on these morphological features, the isolate was identified as *Beauveria bassiana* (Bals.-Criv.) Vuill. [28,29]. Amplification of the internal transcribed spacer (ITS) region yielded a 925 bp fragment. BLASTn analysis revealed >99% identity to *B. bassiana* sequences. In the neighbor-joining phylogenetic tree, strain WZS5 clustered within a well-supported *B. bassiana* clade (bootstrap value = 99%) alongside reference strains SZY2, CZ590, and STB (Figure 2).

### 3.2. Effects of Temperature on the Biological Characteristics of WZS5

**Figure 3:**
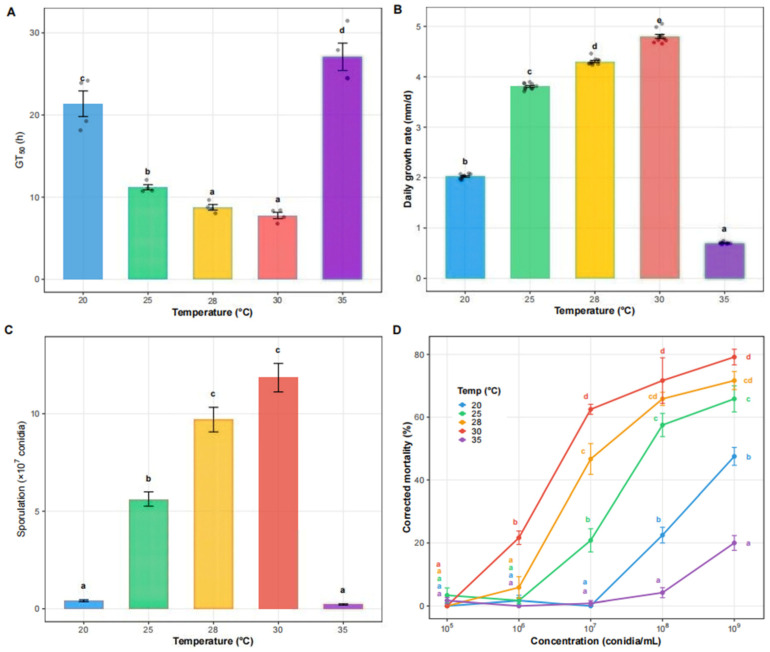
Effects of temperature on biological characteristics and virulence of *B. bassiana* WZS5. (**A**) Median germination time (GT_50_) under five temperatures (20, 25, 28, 30, and 35 °C); (**B**) Mean daily radial growth rate; (**C**) Sporulation yield; (**D**) Corrected mortality of adult *Z. cucurbitae* at different conidial concentrations under five temperatures (20, 25, 28, 30, and 35 °C). Bars represent mean ± SE. Different letters indicate significant differences (*p* < 0.05).

Temperature significantly affected the median germination time (GT_50_) (χ^2^ = 17.89, df = 4, *p* = 0.001), mean daily radial growth rate (χ^2^ = 47.06, df = 4, *p* < 0.001), and sporulation yield (χ^2^= 43.77, df = 4, *p* < 0.001). GT50 was significantly longer at 35 °C and 20 °C than at the other temperatures, while no significant difference was observed between 28 °C and 30 °C (8.1 and 7.7 h, respectively), and both values were significantly shorter than that at 25 °C (11.5 h, Figure 3A). Daily radial growth increased from 2.0 mm/d at 20 °C to 4.8 mm/d at 30 °C, then declined to 0.7 mm/d at 35 °C, with all pairwise differences being significant (Figure 3B). Sporulation yield increased from 4.0 × 10^6^ conidia/plate at 20 °C to 1.1 × 10^8^ conidia/plate at 30 °C, then sharply decreased to 2.0 × 10^6^ conidia/plate at 35 °C (Figure 3C). Yields did not differ significantly between 28 and 30 °C (*p* = 0.376) or between 20 and 35 °C.

### 3.3. Virulence Bioassay as Affected by Temperature and Conidial Concentration

Temperature significantly affected corrected mortality at all tested conidial concentrations except the lowest one (1 × 10^5^ conidia mL^−1^: χ^2^ = 4.00, *p* = 0.406; 1 × 10^6^ to 1 × 10^9^ conidia mL^−1^: χ^2^ =15.47–18.57, *p* < 0.01) (Figure 3D). Mortality increased with conidial concentration. At concentrations from 1 × 10^6^ to 1 × 10^9^ conidia mL^−1^, 30 °C consistently resulted in the highest mortality, followed by 28 °C and 25 °C, whereas mortality was lower at 20 °C and 35 °C. LC_50_ values were lowest at 30 °C (1.32 × 10^7^ conidia mL^−1^) and 28 °C (4.61 × 10^7^ conidia mL^−1^), increased at 25 °C (1.06 × 10^8^ conidia mL^−1^), and were substantially higher at 20 °C (1.49 × 10^9^ conidia mL^−1^) and 35 °C (8.64 × 10^9^ conidia mL^−1^) (Table 1). Similarly, at 1 × 10^8^ conidia mL^−1^, LT_50_ was shortest at 30 °C (5.28 days) and 28 °C (7.40 days), followed by 25 °C (12.04 days), whereas it was much longer at 20 °C (126.68 days) (Table 2). At 35 °C, LT_50_ could not be estimated at 1 × 10^7^ and 1 × 10^8^ conidia mL^−1^ because mortality did not reach 50% during the observation period. However, at 1 × 10^9^ conidia mL^−1^, LT_50_ was estimated at 83.21 days. LT_50_ values for the lower concentration treatments (1 × 10^5^ and 1 × 10^6^ conidia mL^−1^) were not included because these concentrations did not produce sufficient cumulative mortality to reach 50% within the observation period, making reliable estimation of median lethal time impossible.

### 3.4. Horizontal Transmission Within the Target Population

**Figure 4:**
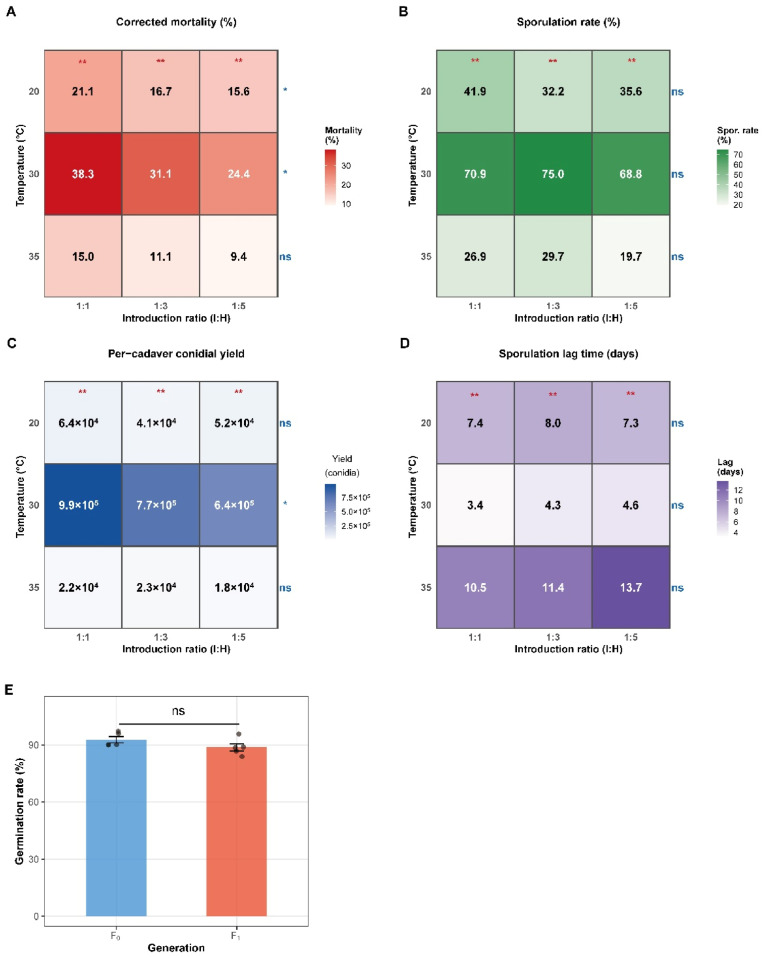
Horizontal transmission, cadaver sporulation, and conidial viability of *B. bassiana* WZS5 under different temperatures and introduction ratios (I:H). (**A**) Corrected mortality in mixed populations; (**B**) Cadaver sporulation rate; (**C**) Per-cadaver conidial yield; (**D**) Sporulation lag time (interval from death to visible mycelial growth); (**E**) 24 h germination rates of primary (F_0_) and secondary (F_1_) conidia. Heatmap values represent treatment means. Asterisks indicate significant differences among temperatures within the same I:H ratio; “ns” indicates no significance.

Corrected mortality varied with both temperature and I: H ratio, and the highest mortality was observed at 30 °C under the 1: 1 ratio (38.3%) (Figure 4A). Temperature significantly affected corrected mortality at all tested ratios (1: 1: χ^2^ = 12.05, *p* = 0.002; 1: 3: χ^2^ = 11.42, *p* = 0.003; 1: 5: χ^2^ = 11.44, *p* = 0.003). Conversely, the I: H ratio significantly influenced mortality at 20 °C (χ^2^ = 7.05, *p* = 0.030) and 30 °C (χ^2^ = 8.95, *p* = 0.011), but not at 35 °C (χ^2^ = 4.70, *p* = 0.095). Under permissive temperatures, the 1: 1 ratio generally resulted in higher mortality than the 1:5 ratio (Figure 4A).

Cadaver sporulation traits were also temperature-dependent. Sporulation rate was highest at 30 °C across all I:H ratios, reaching 75.0% under the 1:3 ratio (Figure 4B). Per-cadaver conidial yield was greatest at 30 °C, with the highest value recorded at the 1: 1 ratio (9.9 × 10^5^ conidia/cadaver) (Figure 4C). In contrast, sporulation lag time was shortest at 30 °C and longest at 35 °C, ranging from 3.4 to 4.6 days at 30 °C and from 10.5 to 13.7 days at 35 °C (Figure 4D). The 24 h germination rate of F_1_ conidia (88.6%) did not differ significantly from that of F_0_ conidia (90.5%) (Wilcoxon rank sum test: W = 22.0, *p* = 0.060; Figure 4E).

### 3.5. Construction of a Temperature-Dependent SIR Epidemiological Model

**Figure 5:**
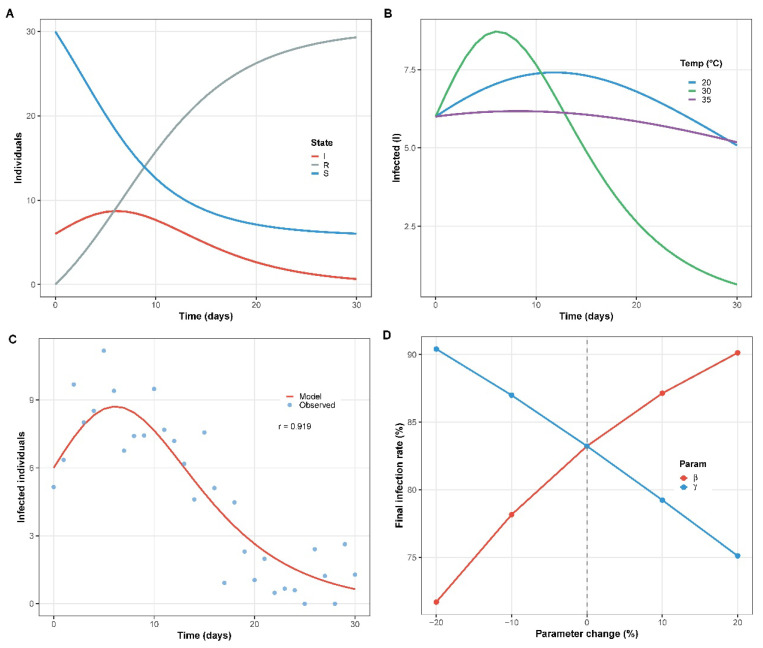
Temperature-dependent SIR model simulations for the *B. bassiana* WZS5-*Z. cucurbitae* system. (**A**) Temporal dynamics of susceptible (S), infected (I), and removed (R) individuals over 30 days; (**B**) Infection dynamics under three temperatures (20, 30, and 35 °C); (**C**) Model-predicted vs. observed infection dynamics (Pearson’s r = 0.919); (**D**) Sensitivity analysis of transmission rate (*β*) and removal rate (*γ*) effects on final infection rate. Dashed line indicates baseline values.

Over the 30-day simulation period, the number of susceptible healthy individuals (S) declined continuously, the number of infected and infectious individuals (I) increased initially and then decreased after reaching a peak, and the number of individuals removed by disease-induced mortality (R) increased (Figure 5A). Model simulations differed markedly among temperatures (Figure 5B). Transmission progressed most rapidly and reached the highest infection peak at 30 °C, was intermediate at 20 °C, and was strongly suppressed at 35 °C. These patterns were consistent with the estimated basic reproduction number (R_0_), which was highest at 30 °C (1.90), followed by 20 °C (1.67) and 35 °C (1.33) (Table 3). Predicted and observed infection dynamics showed strong agreement (Pearson’s r = 0.919; Figure 5C). Sensitivity analysis indicated that the final epizootic size was highly sensitive to both the transmission rate (*β*) and the removal rate (*γ*) (Figure 5D).

## 4. Discussion

In agroecosystems, the use of entomopathogenic fungi is an important component of integrated pest management. However, previous evaluations have largely focused on absolute virulence under controlled conditions, and few studies have integrated environmental temperature, fungal physiological traits, and host population-level epidemiological dynamics into a single quantitative framework. As a result, many biocontrol strains that perform well in the laboratory show considerable variation in efficacy under complex field microclimates, particularly the high temperatures common in tropical regions. To address this practical challenge, we isolated an indigenous *B. bassiana* strain, WZS5, from a high-temperature cucurbit field. In addition to characterizing its temperature-dependent pathogenicity, we combined horizontal transmission experiments with a temperature-driven SIR model to assess the biocontrol potential of this isolate against *Z. cucurbitae* at the population level, thereby providing a quantitative basis for biological control in tropical and subtropical regions.

A key finding of this study is that the indigenous isolate WZS5 displayed high-temperature fitness and may be well suited to tropical environments. Most commercial strains of *B. bassiana* have optimal growth temperatures in the range of 20–25 °C [30], and their growth is often markedly inhibited when temperatures exceed 30 °C [19]. In contrast, the experimental data obtained here showed that strain WZS5 exhibited its highest conidial germination and vegetative growth at 30 °C, and even under sustained heat stress at 35 °C, it retained a basic level of germination and limited sporulation capacity. The thermal tolerance range of an entomopathogenic fungus is generally thought to reflect long-term natural selection imposed by the host and local climatic environment [31]. The strong metabolic performance of WZS5 at 30 °C may provide an important physiological basis for its field application during the tropical summer and autumn.

The thermal adaptability of individual-level physiological processes directly influences fungal pathogenicity against the host [18]. In this study, both the LC_50_ and LT_50_ had their lowest values at 30 °C, indicating optimal virulence within the tested temperature range. Host mortality tended to be higher at temperatures that also promoted greater germination rate and radial growth. From the perspective of infection mechanisms, fungal penetration of the insect cuticle depends on rapid conidial adhesion and germination [12]. At 30 °C, WZS5 reached 50% germination in only 7.7 h, and this rapid process may allow the pathogen to breach the cuticular barrier before the host fully activates immune defenses such as hemocyte phagocytosis and melanization. This interpretation is consistent with the findings reported by Otim et al. [32] (2020) for *Bactrocera dorsalis*. In contrast, the marked decline in virulence observed at both 35 °C (maximum mortality of only 12.5% at the highest concentration) and 20 °C may be attributed to restricted fungal growth under unfavorable temperatures.

Beyond individual-level mortality, epizootic development within a population depends on the ability of infected cadavers to generate secondary inoculum and on host contact behavior. At 30 °C, WZS5 exhibited strong horizontal transmission, resulting in a 30-day cumulative mortality of 62.3% at an infection ratio of 1:1. This was supported by the high cadaver sporulation rate (around 70.0%) and large per-cadaver conidial yield at this temperature, combined with a sporulation lag time of only 4.0 days. Temperature effects on secondary sporulation from fungal-killed cadavers are known to be a major determinant of epizootic scale [33]. In addition, the stable biological performance of F_1_ conidia, with a 24 h germination rate comparable to that of F_0_ conidia, further supports the potential for secondary infection cycles. Considering the gregarious feeding, resting, and frequent courtship and mating behaviors characteristic of tephritid flies [22,34], infected individuals or sporulating cadavers may act as localized infection sources that facilitate continued spread within the population. At 35 °C, however, the greatly reduced per-cadaver conidial yield and extended lag time of 12.0 days would likely weaken this transmission chain.

The temperature-dependent SIR model constructed in this study linked these biological findings to predicted transmission dynamics that may inform field application. At 30 °C, the basic reproduction number (R_0_) reached 1.90, suggesting that the pathogen has the potential for self-sustaining spread within the host population [24,25]. This result has practical implications for IPM: because R_0_ > 1, complete high-dose coverage may not always be necessary under suitable environmental conditions. Model simulations further suggested that even a low initial infection level could lead to substantial population suppression over 30 days through horizontal transmission alone. In addition, sensitivity analysis indicated that the effective transmission coefficient (*β*) was a key parameter governing epizootic development. This suggests that, besides timing fungal applications during ecological windows when daily mean temperatures are between 25 and 30 °C, combining treatments with plant volatile attractants or food-based lures [35] to increase the aggregation frequency of *Z. cucurbitae* (i.e., increasing *β*) may improve control outcomes at lower conidial doses.

It should be acknowledged that, as a baseline study conducted under controlled conditions, this work has certain ecological limitations. For instance, achieving the high efficacy observed through horizontal transmission alone may be difficult in open field environments. The confined physical space of laboratory cages likely increased contact frequency between healthy individuals and infection sources, potentially leading to an overestimation of the transmission coefficient in the model. In addition, intense ultraviolet (UV-B) radiation under natural conditions has been shown to substantially reduce the viability of exposed conidia [36,37,38], and climatic variables such as rainfall [39] were not included in the current model. Therefore, future studies should conduct large-scale field validation in natural orchards and incorporate spatial heterogeneity and climatic stressors into the model.

## 5. Conclusions

This study examined the interaction dynamics between the indigenous *B. bassiana* strain WZS5 and *Z. cucurbitae* across a range of temperatures and provided a quantitative epidemiological analysis of this host–pathogen system. The results indicated that strain WZS5, originally isolated from a microhabitat in Hainan Province, has favorable thermotolerant traits. At 30 °C, rapid conidial germination and active physiological metabolism corresponded to the lowest median lethal concentration and the shortest lethal time. More importantly, this strain was able to efficiently convert fungal-killed cadavers into secondary inoculum sources and achieve horizontal transmission through host contact within the population. The temperature-dependent SIR model showed that, under favorable conditions, even a low initial infection rate may sustain pathogen spread within the host population. Taken together, these findings suggest that *B. bassiana* WZS5 has potential not only as a direct mycoinsecticide but also as a longer-term ecological regulator of field populations. The predictive model developed in this study may provide a useful basis for designing targeted and cost-effective integrated management strategy for *Z. cucurbitae* in tropical and subtropical regions.

## Figures and Tables

**Table 1:** LC_50_ of *B. bassiana* against *Z. cucurbitae* and probit regression parameters at different temperatures.

Temperature (°C)	LC_50_ (Conidia mL^−1^)	Lower 95% CI	Upper 95% CI	Intercept	Slope	Probit Formula
20	1.49 × 10^9^	7.44 × 10^8^	2.98 × 10^9^	−1.7939	0.7407	Probit (p) = −1.7939 + 0.7407 × log10(conc)
25	1.06 × 10^8^	7.10 × 10^7^	1.58 × 10^8^	−1.2413	0.7778	Probit (p) = −1.2413 + 0.7778 × log10(conc)
28	4.61 × 10^7^	3.07 × 10^7^	6.93 × 10^7^	−0.3422	0.6971	Probit (p) = −0.3422 + 0.6971 × log10(conc)
30	1.32 × 10^7^	8.75 × 10^6^	1.99 × 10^7^	0.4031	0.6455	Probit (p) = 0.4031 + 0.6455 × log10(conc)
35	8.64 × 10^9^	2.43 × 10^9^	3.08 × 10^10^	−2.1935	0.7239	Probit (p) = −2.1935 + 0.7239 × log10(conc)

**Table 2:** LT_50_ of *B. bassiana* against *Z. cucurbitae* and probit regression parameters at different temperatures and conidial concentrations. Concentrations at which LT_50_ could not be calculated due to insufficient mortality were not listed.

Temperature (°C)	Concentration (Conidia mL^−1^)	LT_50_ (Days)	Lower 95% CI	Upper 95% CI	Intercept	Slope	Probit Formula
20	1 × 10^8^	126.68	51.86	309.40	2.5474	1.1664	Probit (p) = 2.5474 + 1.1664 × log10(time)
20	1 × 10^9^	24.11	19.71	29.50	3.2353	1.2767	Probit (p) = 3.2353 + 1.2767 × log10(time)
25	1 × 10^7^	132.69	50.02	351.99	3.0737	0.9074	Probit (p) = 3.0737 + 0.9074 × log10(time)
25	1 × 10^8^	12.04	10.29	14.09	3.6541	1.2456	Probit (p) = 3.6541 + 1.2456 × log10(time)
25	1 × 10^9^	7.07	5.68	8.81	4.0921	1.0685	Probit (p) = 4.0921 + 1.0685 × log10(time)
28	1 × 10^7^	23.96	19.32	29.72	3.3624	1.1871	Probit (p) = 3.3624 + 1.1871 × log10(time)
28	1 × 10^8^	7.40	6.19	8.84	3.8618	1.3098	Probit (p) = 3.8618 + 1.3098 × log10(time)
28	1 × 10^9^	4.43	3.31	5.93	4.3258	1.0431	Probit (p) = 4.3258 + 1.0431 × log10(time)
30	1 × 10^7^	11.13	9.54	12.98	3.6088	1.3295	Probit (p) = 3.6088 + 1.3295 × log10(time)
30	1 × 10^8^	5.28	4.30	6.50	4.0150	1.3624	Probit (p) = 4.0150 + 1.3624 × log10(time)
30	1 × 10^9^	3.20	2.23	4.59	4.4945	1.0013	Probit (p) = 4.4945 + 1.0013 × log10(time)
35	1 × 10^9^	83.21	43.20	160.28	2.9482	1.0685	Probit (p) = 2.9482 + 1.0685 × log10(time)

**Table 3:** Estimated basic reproduction number (R_0_) and 95% confidence intervals from the temperature-dependent SIR model.

Temperature (°C)	R_0_ (Mean)	Lower 95% CI	Upper 95% CI
20	1.67	1.23	2.25
30	1.90	1.40	2.57
35	1.33	0.99	1.80

## Data Availability

Experimental data are available from the corresponding author on request.

## Abbreviations

The following abbreviations are used in this manuscript:

SIR	Susceptible–Infected–Removed
ITS	Morphological and molecular
RH	relative humidity
PDA	potato dextrose agar
F_0_	primary
F_1_	secondary
R_0_	the basic reproduction number
